# The Charity Organisation Society and the Hospitals

**Published:** 1897-05-22

**Authors:** 


### The Institutional Workshop.

### THE CHARITY ORGANISATION SOCIETY AND THE HOSPITALS.

The hospitals of London have nothing to thank the Charity Organisation Society for, but rather the contrary. Although hospital authorities as a class, like other people, have aided the Charity Organisation Society in many ways, the promoters of that organisation by their action have used such influence as they possess to destroy, rather than to build up, the voluntary system of hospital support. In other words, whether intentional or otherwise, the policy of the Charity Organisation Society has persistently been to add to the difficulty of responsible managers in raising the necessary funds for hospital support. No sooner was the Prince of Wales's Hospital Fund announced than Mr. C. S. Loch, in the columns of *The Times*, in the publications of the Charity Organisation Society, in certain magazines, and elsewhere, used his utmost endeavours to prevent that Fund from being successful. It has, therefore, created some astonishment that Mr. Loch should now be sending round to the hospitals, on behalf of the Charity Organisation Society, a petition to H.R.H. the Prince of Wales, and should ask the hospital authorities to be kind enough to sign it, with the view, apparently, of strengthening Mr. Loch's hands and the hands of his society in opposing the voluntary system of hospital support. It was noted at the time that the petition promoted by the Charity Organisation Society in favour of a central board was conspicuous from the circumstance that it contained very few names of those who had any right to speak with authority on the subject. We cannot, therefore, suppose that the hospitals are likely to co-operate with Mr. Loch on the present occasion, but that they will prefer to manage their own business in their own way, and if H.R.H. the Prince of Wales is to be approached at all, no doubt the hospitals will combine to prepare their own petition and to address him direct on their own behalf. It is to be regretted that the action of the Charity Organisation Society in this matter should exhibit so little self-respect on the part of its chief promoters. Most people have reason to know the better side of the Charity Organisation Society's work, but there has been of late too much evidence of the restless endeavour which animates its present council in the direction of interference with accredited and approved institutions which are deservedly much stronger than Mr. Loch's society, a society which would have more than enough work to occupy its energies if it were to confine itself to subjects upon which it has a right to speak with authority. Our voluntary hospitals have been managed, on the whole, with singular ability and success by the representatives of the subscribers who provide the money for their maintenance and support. We are glad to know that it is the unanimous feeling of the hospital authorities of London that so strong and valued a friend of our hospitals as H.R.H. the Prince of Wales should not be hampered by the meddlesome interference of outsiders, pestering him with petitions to which they are endeavouring to give a semblance of authority by inducing a few unwary people with a hospital connection to attach their names to them. The hospitals are well content, as the public is well content, to leave H.R.H. the Prince of Wales to determine how and in what manner the money subscribed to his fund shall be distributed, and whom he will invite to aid him in such distribution by their counsel and experience.

